# Asymmetric histone modifications between the original and derived loci of human segmental duplications

**DOI:** 10.1186/gb-2008-9-7-r105

**Published:** 2008-07-03

**Authors:** Deyou Zheng

**Affiliations:** 1 Institute for Brain Disorders and Neural Regeneration, The Saul R. Korey Department of Neurology, Albert Einstein College of Medicine, Rose F. Kennedy Center 915B, 1410 Pelham Parkway South, Bronx, NY 10461, USA

## Abstract

**Background:**

Sequencing and annotation of several mammalian genomes have revealed that segmental duplications are a common architectural feature of primate genomes; in fact, about 5% of the human genome is composed of large blocks of interspersed segmental duplications. These segmental duplications have been implicated in genomic copy-number variation, gene novelty, and various genomic disorders. However, the molecular processes involved in the evolution and regulation of duplicated sequences remain largely unexplored.

**Results:**

In this study, the profile of about 20 histone modifications within human segmental duplications was characterized using high-resolution, genome-wide data derived from a ChIP-Seq study. The analysis demonstrates that derivative loci of segmental duplications often differ significantly from the original with respect to many histone methylations. Further investigation showed that genes are present three times more frequently in the original than in the derivative, whereas pseudogenes exhibit the opposite trend. These asymmetries tend to increase with the age of segmental duplications. The uneven distribution of genes and pseudogenes does not, however, fully account for the asymmetry in the profile of histone modifications.

**Conclusion:**

The first systematic analysis of histone modifications between segmental duplications demonstrates that two seemingly 'identical' genomic copies are distinct in their epigenomic properties. Results here suggest that local chromatin environments may be implicated in the discrimination of derived copies of segmental duplications from their originals, leading to a biased pseudogenization of the new duplicates. The data also indicate that further exploration of the interactions between histone modification and sequence degeneration is necessary in order to understand the divergence of duplicated sequences.

## Abstract 2

A systematic analysis of histone modifications between human segmental duplications shows that two seemingly identical genomic copies have distinct epigenomic properties.

## Background

It is widely recognized that gene duplications, by providing DNA material for evolutionary innovations, have contributed significantly to the complexity of primate genomes. Characterization of the human genome has highlighted the prevalence of segmental duplications (SDs), defined as continuous blocks of DNA that map to two or more genomic locations [1,2]. Previous studies have identified 25,000-30,000 pairs of SD regions (≥90% sequence identity, ≥1 kb), which occupy 5-6% of the human genome and arise primarily from duplication events that occurred after the divergence of the New World and Old World monkeys [2,3]. Detailed characterization of these SDs indicates that several molecular mechanisms might have been involved in the origin and propagation of SDs; in particular, repetitive sequences (for example, Alu elements) seem to have a major role in many segmental duplications [2].

While the contribution of SDs to the architectural complexity of the human genome has been appreciated, the functional and evolutionary consequences of these duplications remain poorly understood. Although studies have begun to define the important roles of SDs in generating novel genes through adaptive evolution, gene fusion or exon exaptation [2,4,5], it remains a mystery how duplicated copies have evolved from an initial state of complete redundancy (immediately after duplications) to a stable state where both copies are maintained by natural selection. On the other hand, recent investigations of duplicated protein coding genes or gene families have provided a glimpse into this important evolutionary process. Those studies have shown that duplicated genes can evolve different expression patterns, leading to increased diversity and complexity of gene regulation, which in turn can facilitate an organism's adaptation to environmental change [6-9]. For example, the expression of yeast duplicated genes appears to have evolved asymmetrically, with one copy changing its expression more rapidly than the other [6].

Initiating from these intriguing observations, the current study explores whether the sequence pairs of SDs are subject to different types and levels of molecular regulation, in particular whether the derived sequences are 'less' functional and are more likely to degenerate. As the majority of SDs are not protein coding, whole genome data unbiased towards genic regions is required to address these questions. Furthermore, such data must have sufficiently high resolution but minimal artifacts, which can often be attributed to high sequence similarity (such as cross-hybridization in microarray analysis), in order to reliably identify distinct signals belonging to each of the two individuals in an SD.

The human genome is organized into arrays of nucleosomes composed of different histone proteins and higher order chromatin structures. Complex profiles of post-translational modifications (for example, acetylation and methylation) of histone proteins are implicated in regulating gene expression and many other important DNA-based biological functions [10-12]. For example, acetylation and H3K4 methylation are often implicated in gene activation while H3K27 methylation and H3K9 methylation are associated with gene repression. As histone modifications can be viewed, to a great extent, as a characteristic of functional chromatin domains, it will be interesting to know how histone modifications between copies of SDs are different. Furthermore, such a study may shed light on the evolution of SDs since histone modifications can modulate the accessibility of SD regions for DNA transcription, replication, and repair [10,13].

This study systematically examined histone modifications in the human SD regions. Using data from a recent chromatin immunoprecipitation and direct sequencing (ChIP-Seq) study [14], the current analysis reveals for the first time that a divergent pattern of modifications exists between the two loci in a pair of SDs, when all SDs are considered collectively. The modifications with an asymmetrical pattern include the methylation of H3K9, H3K27, H3K36, and H3K79. This discovery is very interesting because these modifications have been implicated in a wide range of epigenetic-mediated events, including gene activation, gene repression, and heterochromatin formation [10,14]. Moreover, characterization of SDs emerging after the split of the human and macaque lineages found that the parental copies generally exhibit a higher level of modifications than the derived ones. Intriguingly, parental regions have a greater degree of H3K27me1 and H3K9me1 modifications, but not di- or tri-methylations. Furthermore, the parental loci also differ from the derived loci with respect to gene density, pseudogene density, and the abundance of RNA polymerase II (pol II) association. In short, this study demonstrates that the parental and derived copies of SDs are not functionally identical even though they share ≥90% identity in their primary sequences, suggesting that the descendants in a new genomic environment are more likely the candidates for sequence degeneration or functional innovation.

## Results

### Histone modification data in segmental duplications

The segmental duplications in the human genome were downloaded from the UCSC browser [15,16]. They include 25,914 non-redundant pairs of genomic regions (referred to as SD pairs here) in the released version (hg18) used for this study. The identification of these SDs has been described before [1] and the two sequences in each SD pair have a length of ≥1 kb and share ≥90% sequence identity.

**Figure 1:**
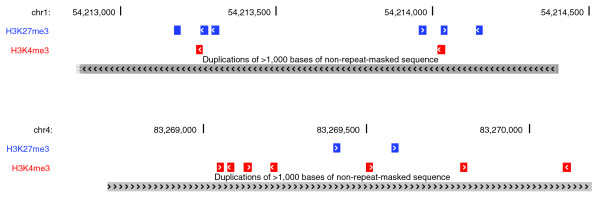
Histone modification ChIP tags in human SDs. A pair of SDs with 91.7% sequence identity was found in chr1:54,212,891-54,214,303 (top) and chr4:83,268,767-83,270,192 (bottom). The top region contained six H3K27me3 and two H3K4me3 ChIP-Seq tags, while the bottom contained two H3K27me3 and seven H3K4me3 tags. Thus, the number of H3K27me3 and H3K4me3 tags per 1 kb are 4.25 and 1.42, respectively, for the top and 1.4 and 4.91 for the bottom region.

Histone modification data were primarily obtained from a recent ChIP-Seq study, which mapped the genome-wide distributions of 20 histone lysine (K) or arginine (R) methylations, as well as H2A.Z, pol II and CTCF (an insulator binding protein) across the human genome [14]. These data are summarized in Table 1, which shows a good number of ChIP-Seq tags (25 nucleotide sequencing reads) from human SDs. Since only tags that can be mapped uniquely to individual SD loci were used, the data in Table 1 indicate that ChIP-Seq can resolve signals from each of the two duplicates in an SD pair. The numbers of tags in SDs, however, decrease as the pairwise similarity within individual SD pairs increases (data not shown). Another set of histone modification data generated by ChIP coupled with paired-end ditags sequencing [17] was also obtained for this study (Table 1). From these two sets of ChIP data, a value measuring the level of a particular nucleosome modification in an SD was derived using a straightforward strategy (Figure 1).

**Table 1:** Summary of source data

Data type	Total data points for the human genome	Points within SDs
H2AZ	7,536,100	152,848
H2BK5me1	8,942,880	184,251
H3K27me1	10,047,279	196,347
H3K27me2	9,070,882	180,054
H3K27me3	8,970,141	176,060
H3K36me1	8,077,127	164,151
H3K36me3	13,572,575	313,579
H3K4me1	11,322,526	213,535
H3K4me2	5,447,902	100,330
H3K4me3	16,845,478	361,316
H3K79me1	10,041,806	213,775
H3K79me2	2,058,068	40,023
H3K79me3	8,114,474	240,709
H3K9me1	9,311,627	170,633
H3K9me2	9,782,127	188,748
H3K9me3	6,348,997	147,639
H3R2me1	9,560,224	208,646
H3R2me2	6,521,560	147,126
H4K20me1	11,015,873	205,009
H4K20me3	5,720,089	370,598
H4R3me2	7,357,597	173,684
Pol II	4,150,378	85,849
CTCF	2,947,043	65,080
		
H3K4me3, ES	478,213	37,413
H3K27me3, ES	257,574	24,480
		
RefSeq genes	18,957	3,366
Duplicated pseudogenes	2,550	1,276
Processed pseudogenes	8,234	2,786
Other pseudogenes	6,809	2,412

In the analyses of histone modifications and transcription factor binding, a data point is a read (that is, tag) from ChIP sequencing. The third column lists the numbers of ChIP tags (or genes, or pseudogenes) within the human SDs.

### Asymmetric profiles of histone modifications in the two regions of segmental duplication

To assess whether two copies of an SD pair exhibit different levels of histone modifications, this study first conducted a paired *t*-test with the null hypothesis that there is no difference. The Wilcoxon signed rank test was also performed to address a concern that ChIP tag differences between the two loci in SD pairs might not distribute normally. The two statistical tests yielded similar results and, therefore, only *t*-test data are discussed. After adjusting multiple testing by the Bonferroni method, 7 of the 20 histone marks showed a difference (adjusted *p *< 0.001; Table 2, all SDs), which include H3K9me2, H3K36me1, H3K79me1, H3R2me1 and the three states of H3K27 methylation. The original ChIP-Seq study also probed the bindings of CTCF and pol II, but the tags for them were distributed between the two loci of SDs without a bias. Similar analysis of the data from human stem cells [17] further indicated that histone modifications are asymmetric between the two copies of SDs (Table 2).

**Table 2:** Statistics for ChIP tag differences in the two copies of human SDs

	All SDs (n = 25,914)		Post-macaque SDs (n = 1,646)						
		
Factors	Paired *t*-test *p*-values	Wilcoxon signed rank test *p*-values	Mean of parental	Standard deviation of parental	Mean of derivative	Standard deviation of derivative	Paired *t*-test *p*-values	Mean of difference	Wilcoxon signed rank test *p*-values
H2AZ	**3.64E-05**	2.86E-07	1.319	2.388	1.114	1.987	5.51E-03	0.205	1.58E-03
H2BK5me1	2.92E-01	2.32E-02	2.600	6.582	1.224	3.237	**1.49E-15**	1.377	2.20E-16
H3K27me1	**2.12E-05**	5.92E-03	2.147	2.415	1.250	1.786	**2.20E-16**	0.897	2.20E-16
H3K27me2	**9.71E-10**	1.74E-11	1.526	1.670	1.355	1.668	5.60E-04	0.170	5.08E-05
H3K27me3	**2.20E-16**	4.60E-14	1.492	1.727	1.460	2.003	6.09E-01	0.031	2.95E-01
H3K36me1	**1.48E-05**	2.28E-10	1.533	1.392	1.242	1.638	**8.66E-10**	0.291	2.20E-16
H3K36me3	1.29E-02	1.57E-05	3.755	6.347	1.796	3.027	**2.20E-16**	1.959	2.20E-16
H3K4me1	3.63E-01	8.01E-03	2.700	6.895	1.139	2.741	**2.20E-16**	1.562	2.20E-16
H3K4me2	8.76E-01	9.66E-06	1.354	2.290	0.651	1.458	**2.20E-16**	0.703	2.20E-16
H3K4me3	6.68E-01	7.46E-11	4.144	16.473	1.987	6.256	**7.06E-07**	2.157	4.44E-16
H3K79me1	**6.49E-12**	2.20E-16	1.911	1.644	1.484	1.781	**2.20E-16**	0.427	2.20E-16
H3K79me2	3.12E-02	1.26E-02	0.476	0.520	0.356	0.499	**1.94E-08**	0.120	3.45E-11
H3K79me3	9.62E-04	2.06E-06	1.823	2.595	1.671	2.941	5.76E-02	0.153	3.76E-05
H3K9me1	2.42E-02	6.41E-04	2.262	3.827	1.046	2.171	**2.20E-16**	1.215	2.20E-16
H3K9me2	**1.67E-07**	3.73E-14	1.618	1.865	1.489	1.936	1.88E-02	0.130	5.80E-03
H3K9me3	2.40E-03	3.90E-06	1.380	2.323	1.357	2.357	7.25E-01	0.023	3.61E-01
H3R2me1	**2.19E-05**	4.41E-09	1.878	1.751	1.440	1.866	**2.20E-16**	0.438	2.20E-16
H3R2me2	4.60E-01	4.41E-01	1.292	1.263	1.079	1.500	**1.40E-06**	0.213	1.18E-11
H4K20me1	5.72E-04	6.77E-05	4.865	18.476	1.257	5.664	**9.85E-13**	3.608	2.20E-16
H4K20me3	3.23E-01	5.78E-01	1.687	6.408	1.953	5.677	1.26E-01	-0.266	2.03E-01
H4R3me2	2.16E-01	5.43E-01	1.439	1.404	1.165	1.666	**1.91E-10**	0.275	6.66E-16
Pol II	4.24E-01	2.45E-04	1.538	4.476	0.507	0.812	**6.47E-16**	1.031	2.20E-16
CTCF	9.31E-02	6.45E-05	0.757	1.560	0.521	1.226	**1.41E-06**	0.236	2.20E-16
									
H3K4me3, ES	**0.0008**	0.294	5.673	9.20	1.423	4.89	**2.30E-06**	4.25	1.91E-10
H3K27me3, ES	0.0024	7.63E-06	2.203	3.255	1.958	3.314	0.71	0.245	0.534

The *p*-values are before adjustment for multiple testing; statistically significant results (by *t*-test) are in bold.

### Higher level of histone modifications in the parental versus derivative loci of segmental duplications

**Figure 2:**
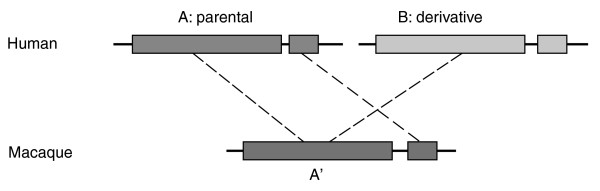
A cartoon illustrating the method used here for identifying post-macaque SDs based on chromosomal synteny. Using the liftOver tool [29] from the UCSC genome browser group, a pair of human SDs (A and B) is mapped to the same location (A') in the macaque genome. A and B (large block) are thus considered the product of an SD event that occurred after the split of human from macaque lineages. Then 1 kb sequences (small block) adjacent to A or B were aligned to the macaque genome. If only the sequence next to A was mapped next to A', then A is designated as the parental copy and B as the derivative.

Next, I investigated whether the asymmetry is due to uneven histone modifications between the parental and the derivative regions. Although it has been previously found that two duplicated genes can evolve distinct functions, no systematic study to date has addressed which copy diverges away from its ancestral function. Unfortunately, current SD data do not contain the directionality of duplications, and accurate identification of duplication direction remains a challenge. This study thus adopted a strategy that was recently applied to identify ancestral duplication loci [18]. As illustrated in Figure 2, this approach relies largely on chromosomal synteny (that is, order of sequences on a chromosome) and uses macaque as an outgroup species to assign duplication directions for SDs. It produced more accurate parental-derivative relationships than other methods that were based entirely on mutual best hits established by sequence comparison, because a synteny-based strategy is more appropriate for identifying evolutionarily equivalent sequences in mammalian genomes. Macaque was chosen here because its genome has been sequenced and the average human-macaque sequence identity is approximately 93% [19], which is near the 90% used in identifying SDs. The current approach is not meant to systematically assign SD directions but to select SDs for subsequent analyses, because it can be applied only to SDs that arose after the split of human and macaque lineages. Nevertheless, it was able to determine the parental-derivative relationship for 1,646 SD pairs, referred to here as post-macaque SD pairs.

A paired *t*-test for these 1,646 pairs of post-macaque SDs revealed that 14 histone modifications are different between parental sequences and their derivative copies, including H3K36me1, H3K79me1, H3R2me1 and H3K27me1, which also showed asymmetries in the above analysis of all SDs (Table 2). In particular, histones in the parental loci exhibited a higher level of mono-methylation of H3K27 and H3K9 than those in the derivative regions (Table 2), but no difference was detected for di- and tri-methylations. Data from stem cells further supported a difference in H3K4me3 but no difference in H3K27me3. Interestingly, pol II and CTCF were relatively abundant in the parental versus the derivative loci. Noticeably, the analysis of post-macaque SDs yielded a list of histone marks that is quite different from what was obtained for all SDs (Table 2), suggesting that duplication direction is an important factor to include in examining disparate features of duplicated genes.

**Figure 3:**
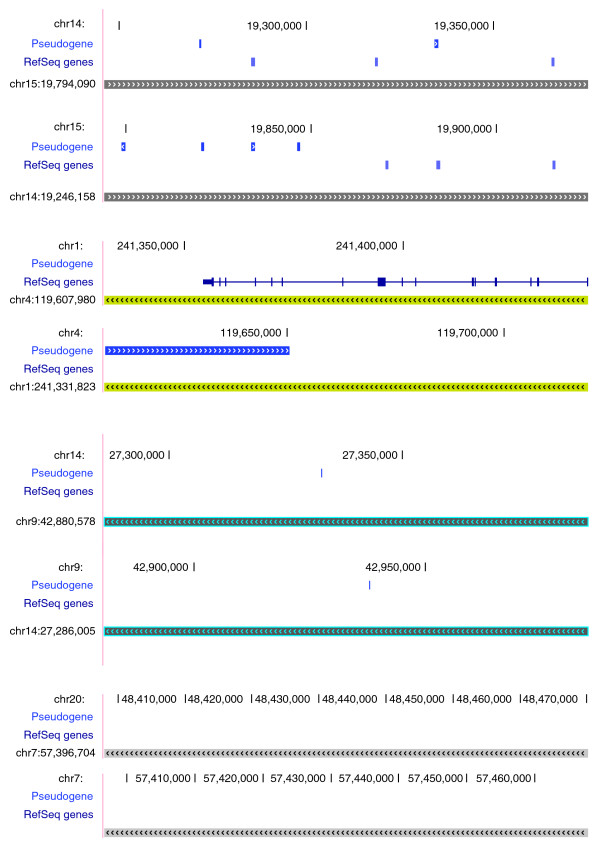
Gene and pseudogene annotations in four pairs of human SDs with known duplication directions. The parental locus of each pair is depicted first, followed immediately by its derivative.

**Figure 4:**
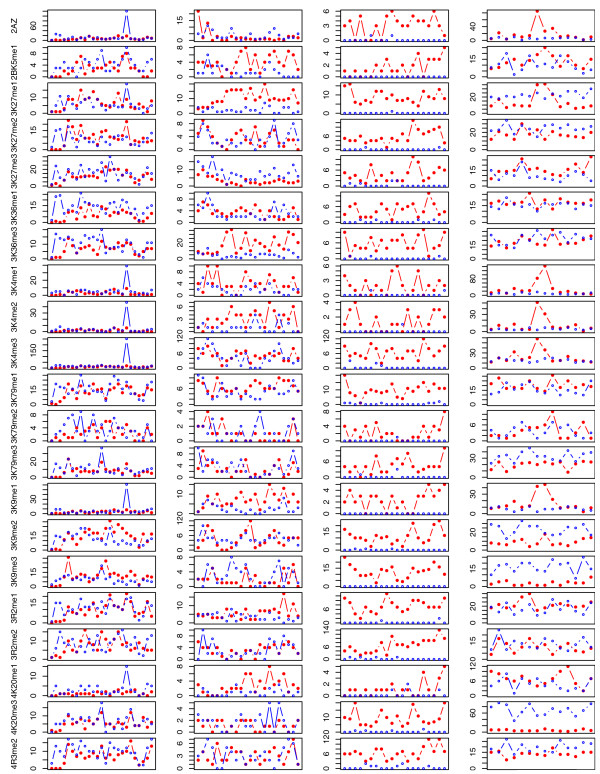
Pattern of histone modifications for the four SD pairs in Figure 3, ordered left to right to match their order from top to bottom in Figure 3. Each point represents the number of ChIP-Seq tags in a 5 kb genomic region, with red for parental and blue for derivative SDs. Horizontal axes are the position relative to the 5' end of a parental locus. Data for a derivative region is ordered with respect to its parent.

The distribution of ChIP-Seq tags was further examined for human segmental duplications with known duplication directions. Previously, Eichler's research group have determined the duplication directions of nine human SDs by comparative fluorescent *in situ *hybridization (FISH), using genomic sequences in a human derivative locus as a probe against chromosomes from an outgroup primate species [18]. Four of those nine pairs are depicted in Figure 3. Analysis of ChIP-Seq data found that the levels of histone modifications were in fact quite biased between the two loci of most of these SD pairs. Especially, the parental regions were statistically higher for the following methylations: H2BK5me1, H3K4me2, H3K9me1, H3K27me1, H3K36me3, and H3K79me1. Mono-methylation seems to make up the bulk of the differences. Figure 4 shows the distributions of ChIP-Seq tags for four of these nine SDs.

The paired *t*-test described above, in principal, compared the sums of ChIP tags in the two copies of an SD pair, but overlooked the intra-SD tag distributions. Thus, a non-statistical method was developed to address this through analyzing ChIP tags in a set of large SDs (>15 kb). Briefly, these SDs were first divided into non-overlapping blocks. Then, for each pair of SDs, one locus was determined to have a higher level of a histone modification if at least two-thirds of its blocks contained more tags of this modification than the corresponding blocks of the other locus. The results not only show that SD loci with a greater degree of modification were three to six times more likely to be parental (Table 3), but also indicated that asymmetry often existed across an SD locus, rather than in one or few narrow sub-regions. Interestingly, all modifications exhibited some degree of asymmetry by this measurement. The second and third examples in Figures 3 and 4 illustrate such a pattern of asymmetrical modifications of histones.

**Table 3:** Numbers of large (>15 kb) post-macaque SDs with higher histone modifications in either parental or derivative loci

Factors	Higher in parental loci	Higher in derivative loci
H2AZ	68	23
H2BK5me1	92	15
H3K27me1	96	17
H3K27me2	85	19
H3K27me3	85	29
H3K36me1	97	14
H3K36me3	90	23
H3K4me1	83	14
H3K4me2	67	12
H3K4me3	93	15
H3K79me1	87	19
H3K79me2	37	9
H3K79me3	82	23
H3K9me1	84	16
H3K9me2	83	24
H3K9me3	73	15
H3R2me1	103	19
H3R2me2	93	18
H4K20me1	81	14
H4K20me3	72	27
H4R3me2	88	18
Pol II	57	15
CTCF	51	13

### More parental loci of segmental duplications exhibit 'peak' signals of histone modifications

**Figure 5:**
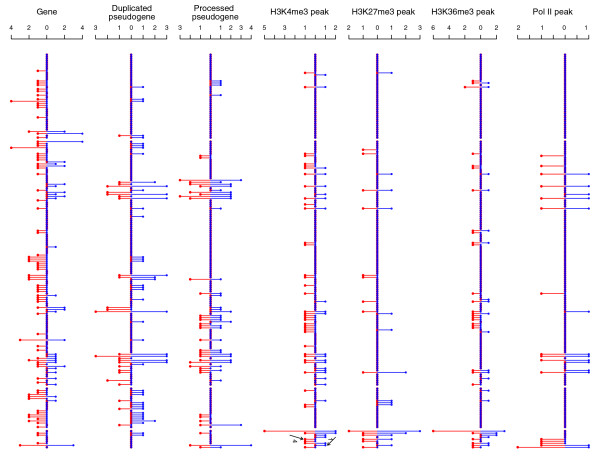
The peaks of ChIP-Seq signals in large post-macaque SDs. The numbers of peaks (see Materials and methods) for H3K4me3, H3K27me3, H3K36me3, and pol II are plotted for each of the large SD pairs (from top to bottom), along with the numbers of genes and pseudogenes. The numbers on the left (red) and right (blue) are for parental and derivative SDs, respectively. The H3K4me3 peaks in the first and forth example of Figure 4 are marked by an arrow and labeled with 1 and 4, respectively.

'Peaks' of histone modifications in these large SD pairs were also studied. In agreement with the above observations, the peaks of ChIP-Seq signals were more frequently located within the parental SDs than the derivative SDs, especially for the three marks H3K4me3, H3K9me1, and H2A.Z, which have been previously shown to be enriched in promoters [14]. Data for H3K4me3, H3K27me3, and H3K36me3 are shown in Figure 5 because these methylations are known characteristic marks of promoters and transcribed regions, with H3K4me3 correlating with active genes and H3K27me3 relatively enriched at silent promoters [10,12,14,20]. As shown (Figure 5), SDs with an H3K4me3 peak were 1.5 times more likely to be parental. Such a bias, however, was not detected for H3K27me3. Only approximately 50% of either parental or derivative SDs with H3K4me3 peaks contained genes, suggesting that more functional elements (including novel protein coding and non-coding genes) are yet to be annotated in the human SDs. Interestingly, 9 of the 16 parental SDs versus 4 of the 16 derivative SDs with H3K27me3 peaks contained annotated genes, but these numbers were not statistically significant enough to claim that fewer genes in the derived SDs were repressed in CD4^+^ T cells. Parental SDs appeared more likely to have H3K36me3 and pol II peaks; however, those peaks did not seem to co-exist in the same SDs as frequently as expected from the correlation previously reported between H3K36me3 and actively transcribed regions [14,20]. This inconsistency needs to be studied in the future. Additionally, it needs to be mentioned that the known correlations between histone methylations and transcription start sites (TSSs) [14] were observed for the TSSs within SDs, and the patterns for parental SDs and derivative SDs were mostly indistinguishable (data not shown).

In summary, characterization of the pattern of histone modifications by various measurements consistently revealed an asymmetrical pattern of histone modifications, with higher levels biased to the parental regions of SDs, demonstrating that two seemingly 'identical' genomic copies are actually distinct in their epigenomic properties.

### Parental loci of segmental duplications contain more genes but fewer pseudogenes

It has been reported that SDs are generally enriched with genes [2,3]. This is confirmed by the current survey of genes and pseudogenes in human SDs (Table 1); note that SDs occupy approximately 5% of the human genome. Moreover, Table 1 shows that human SDs are more enriched with pseudogenes than genes, as 36.8% of human pseudogenes and 17.8% of human genes are located in SDs (*p *<< 0.001). Duplicated pseudogenes appear more likely to be associated with SDs than processed pseudogenes, as 50% of human duplicated pseudogenes versus 33.8% of processed pseudogenes are in SDs (*p *<< 0.001). This is consistent with the fact that duplicated pseudogenes are generated by gene duplications whereas processed pseudogenes are from retrotranspositions.

A subsequent examination of genes and pseudogenes in the 1,646 post-macaque SDs revealed that 656 parental and 192 derivative loci contain genes (Table 4), while significantly more pseudogenes (all types) are in the derived regions. The numbers of genes and pseudogenes for large SDs are also shown in Figure 5, which clearly illustrates that genes and pseudogenes are enriched in the parental and derived loci, respectively. These data suggest that duplicated sequences in the derived loci are more frequently subject to degeneration and pseudogenization than the parental sequences. It is also possible that duplications yield mostly 'broken' genes in the new locations. However, the combined number of genes and pseudogenes is also higher in the parental SDs. Moreover, when both parental and derived loci were compared to their 'ancestral' locus in the macaque genome (Figure 2), the average sequence identity was 89.8% (±5.9%) and 88.8% (±6.1%) for the parental and derivative, respectively. This difference is statistically significant (*p *= 3e-10), further suggesting a faster degeneration of derived sequences.

**Table 4:** Numbers of post-macaque SD loci with genes or pseudogenes

	Parental	Derivative
RefSeq genes	656 (716)	192 (213)
Duplicated pseudogenes	113 (131)	251 (279)
Processed pseudogenes	161 (219)	269 (331)
Other pseudogenes	124 (143)	209 (232)

For reference, the numbers of genes/pseudogenes are also listed in parentheses as some loci can have more than one gene or pseudogene.

### Pseudogenization and asymmetry in histone modifications

How does the asymmetry in histone modifications relate to gene content and gene death in human SDs? The asymmetry of pol II ChIP tags is certainly consistent with the biased distribution of genes because more pol II tags usually indicate higher degrees of transcriptional activity. This correlation is further supported by the observation that most histone modifications enriched at promoters are higher in parental SDs (Tables 2 and 3).

The asymmetric distribution of genes, however, cannot fully account for the asymmetric profiles of histone modifications described above. Firstly, the asymmetrical pattern remained present, though consisted of fewer marks, when the above *t*-test was restricted to 623 post-macaque SD pairs containing neither genes nor pseudogenes in both loci. The significantly different modifications are H3K9me1, H3K27me1, H3K4me1, H3K4me2, H3K79me1, and H3K79me2. Secondly, analysis of SDs without genes also detected a skew for the histone marks H3K9me1, H3K27me1, H3K79me2, H4K20me3, and the three states of H3K4 methylation. All of these modifications occurred more frequently on the parental loci, except H4K20me3, which was previously found to associate with repressive chromatin [21]. Thirdly, an analysis restricted to 419 SD pairs that did not exhibit a difference in pol II between their two copies (defined as difference of pol II <0.3 tag per kb) found several marks with significant asymmetry, including H3K9me1, H3K27me1, H3K27me2, H3K36me1, H3K36me3, and H3K79me2. It is interesting to see that H3K79me2, which was found without a significant preference toward either active or silent genes [14], shows a difference here. In this analysis, the statistics for pol II is a *p*-value of 0.46.

Gene and pseudogene contents, nevertheless, have an influence on the asymmetrical pattern of epigenomic modifications (Figure 5). Not only did fewer marks exhibit a difference in the characterizations of 'gene-depleted' SDs, but also the pattern was less biased to the parental copies. For example, the difference of mean tag densities was 1.215, 0.897, 1.562, 0.703, and 0.427 for H3K9me1, H3K27me1, H3K4me1, H3K4me2, and H3K79me1, respectively (Table 2). These numbers decreased to 0.461, 0.389, 0.741, 0.271, and 0.357, respectively, for the SD pairs without genes or pseudogenes. In addition, a characterization of SD pairs (n = 103) with genes in both of their loci did not find a modification with a significantly asymmetrical pattern, though a difference was observed for H3K36me3 and H4R3me2 (unadjusted *p*-value < 0.001).

### Shift in the patterns of differences in histone modification as segmental duplications age

Finally, in order to address the dynamics of the above asymmetries during evolution, the post-macaque SDs were split into four groups based on pairwise nucleotide sequence identity of SD pairs (Table 5). The parental and derivative copies of young SDs (sequence identity ≥0.975) exhibited uneven H3K27me1, H3K36me3, H3K9me1, and H4R3me2 modifications. The first two marks were both enriched downstream of transcription start sites [14]. As SD sequences age, more modifications with an asymmetric pattern emerge and then potentially disappear, but differences in H3K27me1 and H3K9me1 modifications persist. Although a difference in gene content was observed across all age groups, this analysis found that as SDs evolve more genes in the derivative loci have been lost, presumably becoming pseudogenes (Table 5). Pseudogenes (of all three types) were always more abundant in the derivative than the parental loci. This is true even for the oldest SDs, though the difference becomes statistically less significant; for example, the means of duplicated pseudogenes were 0.157 and 0.238 for the parental and derivative regions (*p*-value = 0.02), respectively.

**Table 5:** Features with asymmetric distribution between the parental and derivative loci of post-macaque SDs grouped by sequence identity

	Sequence identity			
	
	<0.925 (n = 330)	0.925-0.95 (n = 444)	0.95-0.975 (n = 570)	≥0.975 (n = 302)
Significantly different modifications (paired *t*-test, adjusted *p*-value <0.001)	H3K27me1	H3K27me1	H2BK5me1	H3K27me1
	H3K4me2	H3K36me1	H3K27me1	H3K36me3
	H3K9me1	H3K36me3	H3K36me3	H3K9me1
	H3R2me1	H3K4me1	H3K4me1	H4R3me2
		H3K4me2	H3K4me2	
		H3K79me1	H3K79me1	
		H3K79me2	H3K9me1	
		H3K9me1	H3R2me1	
		H3R2me1	H4K20me1	
				
Genes/pseudogenes (*p*-value <0.001)				
RefSeq genes	0.375/0.087	0.341/0.087	0.367/0.127	0.410/0.191
Duplicated pseudogenes	None	0.089/0.223	0.043/0.135	None
Processed pseudogenes	None	0.238/0.413	0.104/0.367	0.063/0.296
Other pseudogenes	None	0.089/0.253	0.056/0.238	0.055/0.285

The values for genes/pseudogenes are the average number of genes (or pseudogenes) per 1 kb for parental/derivative sequences. Only features with statistical significance are listed.

## Discussion

Duplication of genomic sequence is an important evolutionary process that supplies raw genetic material for architectural as well as functional innovations. Its prevalence has been observed in all three kingdoms of life, with several distinct mechanisms leading to their abundance [2,5,22]. A duplication occurring in a single individual can be fixed or lost in the population, but the most common consequence seems to be the loss of all or part of the newly duplicated sequences through deletion or degeneration. Nonetheless, a novel biochemical function can sometimes arise from the redundant sequences.

The asymmetrical distributions of histone modifications, genes, pseudogenes, and transcription (with pol II as the proxy) between parental and derivative loci of human SDs support that degeneration (or pseudogenization) is more common than innovation (or neofunctionalization) after gene duplications. One important discovery here is the depletion of genes and, conversely, the enrichment of pseudogenes in the derivative loci. This implies either that most duplications are incomplete when occurring - that is, only part of a gene is duplicated to the new location, resulting in a pseudogene at birth - or that deletion plays a large role in disabling the descendant sequences. The former is supported by more non-processed pseudogenes in derivative regions, while the latter is probably related to the difference in the sum of genes and duplicated pseudogenes in the two copies (Table 4), though it may be influenced by incomplete gene annotation in SDs as well. The results suggest that the original copy is evolutionarily constrained to maintain its functional status while the descendant is relatively free to mutate and can eventually become a 'non-functional' sequence. It is kind of amazing to see that an organism can achieve this given that the two copies are seemly identical in their primary sequences. The current report of gene difference is also consistent with a recent finding that core duplicons, the common DNA subunits sharing by multiple SDs, are enriched for genes and spliced expressed sequence tags [18]. Unfortunately, due to the limitation of the current strategy for identifying the direction of duplication, not enough SD data were produced to address precisely the different rates of pseudogenization in the parental and derived loci. This issue will be addressed in the future when more primate genomes are sequenced and improved algorithms are developed for reliably identifying SDs of sequence identity <90%.

The asymmetry of histone modifications can be a direct consequence of more genes and fewer pseudogenes in the parental loci as histone modification is a process often occurring near genes that can lead to either gene activation (for example, H3K4 methylation) or repression (for example, H3K27 methylation). Such a correlation is apparent for H3K4me3 in large SDs (Figure 5). It is also supported by the analysis of SD pairs containing functional genes in both of their loci, whereas almost no modifications exhibited a significantly unsymmetrical pattern. The small sample size, however, could be an issue for generalizing that result.

Alternatively, the current findings may suggest that the chromatins in derivative SDs are looser relative to those in the parental. Under this scenario, the genomic sequences in the derived loci are prone to mutations because of their greater exposure, leading to more pseudogenes in evolution, and the turnover rate of nucleosomes in the derivative regions is higher (that is, exchange faster with free histones), resulting in fewer modified histones being detected experimentally. This can explain why higher levels of various modifications were always seen in the parental SDs. Likewise, loose chromatins are more vulnerable to retrotranspositions; as a result, more processed pseudogenes were inserted into the derived loci of SDs (Tables 4 and 5). Along the same line, it is worth noticing that derived loci containing duplicated pseudogenes often have processed pseudogenes too (Figure 5). Furthermore, this hypothesis is particularly supported by the data from a recent study [23] that mapped nucleosome positions using the Solexa sequencing technique. Analysis of those reads indeed revealed that nucleosomes were relatively depleted (*p *<< 0.001) in the derived SDs.

Other biological processes may have also contributed to the asymmetries reported here. First of all, the derived SDs may have ended up in regions of repressive chromatins. The genome distribution of post-macaque SDs showed that centromeres contained slightly more derivative SDs than parental SDs (data not shown). However, characterization of post-macaque SD pairs (n = 1,313) whose two loci were at least 5 Mb away from heterochromatin regions found essentially the same asymmetrical histone modifications that were observed for all post-macaque SD pairs. For example, the study of those restricted SD pairs also showed that mono-methylations of H3K9 and H3K27 were higher in the parental SDs but not di- and tri-methylations of H3K9 and H3K27. Since H3K27 and H3K9 methylations are often associated with chromatin repression [10,14,20] but they did not exhibit an enrichment in the derived SDs, the impact of repressive chromatin on the observed asymmetry of histone modifications is small but warrants further investigations. On the other hand, these results cannot rule out that histone modifications may have been directly involved in the initial regulation of the descendant sequences by keeping the extra genomic copies in a silent chromatin state (for example, by not modifying histones). Conversely, histone modifications may have facilitated degeneration of the descendant sequences by increasing the accessibility of those DNAs for a greater rate of mutations. Both scenarios are very important for understanding SD evolution; however, they cannot be confidently separated in the current analysis.

In any case, the characterization of either SDs without genes or SDs without pol II asymmetry shows that asymmetrical distributions of several histone modifications were not entirely entangled with gene/pseudogene asymmetries. It is very difficult to really resolve the interaction between sequence degeneration (or pseudogenization) and epigenomic changes, largely because almost all histone marks that have been characterized were investigated in the context of gene expression (either activation or repression). Analysis of post-macaque SDs in different age groups did not help untangle this issue either. If pseudogenization is the cause of asymmetric histone modifications, we would expect to see more asymmetries emerge as SDs age; conversely, we would see asymmetries fade away if they facilitate pseudogenization. The data in Table 5 provide evidence for both or neither, depending on one's interpretation. Further studies are required to address all these questions, and more generally to fully appreciate the potential importance of epigenetic modifications in the initial regulation and subsequent evolution of duplicated sequences. As shown in Figure 4, not all parental loci of SDs exhibited a higher level of histone modification than their derivative regions. Such SD pairs may contain asymmetrical histone acetylations or phosphorylations, or the derivative loci have newly emerging functional elements. In the future, integrated analysis of different types of modifications is certainly necessary as the effect of an individual histone modification is likely context-dependent and cannot simply be referred to as either activating or repressing a chromatin domain [10,11,13,14].

Finally, the asymmetry in histone modifications may be relevant to the established view that divergence in regulatory elements is the first step of functional divergence between duplicated genes. Several previous studies have suggested that duplicated genes often evolve at different rates; for instance, one study found that expression of duplicated genes tends to evolve asymmetrically [6]. The expression of one copy evolves rapidly, likely through changes in its regulation, whereas the other one largely maintains the ancestral expression profile. It will be interesting to see if the changing copy is the parental or the derivative and whether histone modifications are involved in establishing the disparate profile of expression and in facilitating subsequent functional divergence. A separate study has found that retrotransposed genes tend to undergo accelerated evolution relative to their parental genes [24]. The discovery of asymmetrical histone modifications here is consistent with these early results and points to a new direction to explore those early findings.

## Conclusion

This study is important for understanding both the functional influence and evolutionary fate of SDs because it indicates that derivative sequences of SDs become non-functional more often than the originals, as measured by histone modifications, transcription, and density of genes or pseudogenes. This finding is significant because it pinpoints, for the first time, derived sequences as the main locations of divergent evolution between duplicated genomic regions, suggesting that evolution selects a parental locus to maintain its original biological property but allows its derivative sequence to mutate freely, eventually leading to either degeneration or functional innovation.

## Materials and methods

The SD regions were obtained from the UCSC browser [15,16]. The hg18 version contained 51,809 pairs of SDs. After redundant entries were removed, as a pair of segmental duplications was usually listed twice by switching the order of the two regions, 25,914 non-redundant SD pairs were used for this study. RefSeq genes [25] were also downloaded from the UCSC browser and then overlapping transcripts were collapsed into a gene. Human pseudogenes were obtained from the Pseudogene.org database [26]. The identification of these pseudogenes has been described previously [27,28]. Processed and duplicated pseudogenes were separated from the rest, which usually do not contain obvious sequence features of retrotranspositions or exon-intron structures [26,28].

Two sets of histone profiling data were used, one for the human resting CD4^+^ T cells [14] and the other for the human embryonic stem cells [17]. These data (or tags) identified the human genomic regions where modifications of nucleosomes or binding of pol II and CTCF were detected. In both cases, the genomic coordinates of ChIP tags were obtained from the original authors and this study did not re-align ChIP sequencing reads to the human genome. Figure 1 describes the general strategy of counting ChIP tags for individual segmental duplications. For statistical analysis, the number of tags per 1 kb genomic region was used to represent the level of each modification. A similar approach was applied to map genes or pseudogenes into SDs, but a gene or pseudogene was assigned to an SD if it overlaps this SD by at least 1 bp.

Figure 2 illustrates the approach for identifying segmental duplications that arose after the split of human and macaque ancestors. Its principle is chromosomal synteny between the human genome and the macaque genome. Using a very strict criterion, this method recognized 2,654 SD events after the divergence; however, it only resolved the direction of duplications for 1,646 SD pairs. This strategy was designed to only extract (post-macaque) SDs with an easily identifiable direction of duplication.

In order to characterize the distribution of histone modifications within SDs in detail, a non-statistical method was applied to 185 large (>15 kb) post-macaque SD pairs. Each of these SDs was divided into a set of continuous but disjointed blocks (5 kb), which was in turned represented by a vector describing ChIP tags. Thus, the parental vector was P = [p_1_, p_2_, ..., p_m_] and the derivative vector was D = [d_1_, d_2_, ..., d_n_], where P_i _and D_i _were the numbers of ChIP-Seq tags in the i-th block. 1..n was ordered with respect to 1..m, and m = n for most SDs. Let x = y = 0; and for i in 1..b (b be the smaller of m and n), x increased 1 if P_i _> D_i _but y increased 1 if P_i _< D_i_. Then, for each pair of SDs, its parental locus was considered to have a higher level of a histone modification if x > 2/3 * b, otherwise, the derivative locus was higher if y > 2/3 * b. The result of this analysis is shown in Table 3, and the P and D for four pairs of SDs with their duplication directions known are illustrated in Figure 4.

The ChIP-Seq signal 'peaks' in these large SDs were also identified. Many software and algorithms exist for calling peaks from ChIP-Seq reads; however, they were not used here because ChIP-Seq reads in SDs have a distribution quite different from those in non-SD regions (tag density is much lower; Table 1). Instead, a peak here was simply defined as a block (5 kb) with >5 ChIP-Seq reads and the read count was also two standard deviations above the average read in this SD. This method correctly reported the apparent H3K4me3 peaks in the first and forth example of Figure 4. The numbers of such peaks for the 185 large SD pairs are plotted in Figure 5 for H3K4me3, H3K27me3, and H3K36me3 because these three methylations are well characterized in the literature.

## Abbreviations

ChIP-Seq, chromatin immunoprecipitation and direct sequencing; pol II, RNA polymerase II; SD, segmental duplication; TSS, transcription start site.
